# Age-Related Decrease in the Mitochondrial Sirtuin Deacetylase Sirt3 Expression Associated with ROS Accumulation in the Auditory Cortex of the Mimetic Aging Rat Model

**DOI:** 10.1371/journal.pone.0088019

**Published:** 2014-02-04

**Authors:** Lingling Zeng, Yang Yang, Yujuan Hu, Yu Sun, Zhengde Du, Zhen Xie, Tao Zhou, Weijia Kong

**Affiliations:** 1 Department of Otorhinolaryngology, Union Hospital of Tongji Medical College, Huazhong University of Science and Technology, Wuhan, Hubei province, P. R. China; 2 Department of Otorhinolaryngology, Nanshan Affiliated Hospital of Guangdong Medical College, Shenzhen, China; Oregon Health & Science University, United States of America

## Abstract

Age-related dysfunction of the central auditory system, also known as central presbycusis, can affect speech perception and sound localization. Understanding the pathogenesis of central presbycusis will help to develop novel approaches to prevent or treat this disease. In this study, the mechanisms of central presbycusis were investigated using a mimetic aging rat model induced by chronic injection of D-galactose (D-Gal). We showed that malondialdehyde (MDA) levels were increased and manganese superoxide dismutase (SOD2) activity was reduced in the auditory cortex in natural aging and D-Gal-induced mimetic aging rats. Furthermore, mitochondrial DNA (mtDNA) 4834 bp deletion, abnormal ultrastructure and cell apoptosis in the auditory cortex were also found in natural aging and D-Gal mimetic aging rats. Sirt3, a mitochondrial NAD+-dependent deacetylase, has been shown to play a crucial role in controlling cellular reactive oxygen species (ROS) homeostasis. However, the role of Sirt3 in the pathogenesis of age-related central auditory cortex deterioration is still unclear. Here, we showed that decreased Sirt3 expression might be associated with increased SOD2 acetylation, which negatively regulates SOD2 activity. Oxidative stress accumulation was likely the result of low SOD2 activity and a decline in ROS clearance. Our findings indicate that Sirt3 might play an essential role, via the mediation of SOD2, in central presbycusis and that manipulation of Sirt3 expression might provide a new approach to combat aging and oxidative stress-related diseases.

## Introduction

Age-related hearing loss (AHL), also known as presbycusis, is the most common sensory disorder among elderly persons. The decline in hearing sensitivity caused by aging is related to the deterioration of the central and/or peripheral auditory system [1]. The prevailing otolaryngologic approach to the treatment of AHL emphasizes the compensation of peripheral functional deficits, such as hearing aids and cochlear implants. However, the cost of these therapeutic options represent a major barrier to their utilization. Thus, a better understanding of the molecular mechanisms underlying AHL may overcome this obstacle by opening doors to new treatment options, such as drug therapy in the auditory system. Central presbycusis refers to age-related changes in the auditory portion of the central nervous system, which negatively affect auditory perception, speech-communication performance, or both [2]. Age-related dysfunction of the central auditory cortex is well known to affect sound localization [3]. However, the mechanisms involved in central presbycusis remain unclear. Because traditional surgery is not suitable for central presbycusis, illustrating the pathological process and identifying potential therapeutic targets are particularly important.

The free radical theory postulates that accumulation of oxidative damage caused by reactive oxygen species results in aging [4], [5]. ROS are inevitable byproducts of oxidative phosphorylation. Oxidative stress, an imbalance between the generation and elimination of ROS, can oxidatively modify a variety of molecules (DNA, proteins and lipids) in organisms ranging from invertebrates to humans [6]–[9]. mtDNA is highly susceptible to oxidative damage, and mtDNA mutations accompany normal aging [10], [11]. Thus, the common mtDNA deletion (the CD, 4977 bp deletion in human, 4834 bp deletion in rats) has been used as a biomarker for aging [12]–[15]. Oxidative stress has been reported to be involved in the pathogenesis of age-related degenerative disorders, such as Alzheimer's disease, Parkinson's disease and Huntington's disease [16]–[18]. Moreover, oxidative stress has also been postulated to play a causal role in age-related hearing loss. Several studies have shown that ROS are generated in cochleae exposed to high-intensity noise [19]. Mice lacking antioxidant enzymes demonstrate enhanced age-related cochlear hair cell loss and susceptibility to noise-induced hearing loss [20], [21]. Moreover, oxidative protein damage increases with age in the cochleae of CBA mice [22]. Previous studies from our laboratory have shown that oxidative stress induced by overdoses of D-Gal *in vivo* can mimic normal aging in rats [15], [23], [24]. However, the molecular mechanisms by which age and D-Gal increase oxidative stress remain unknown.

Sirtuins are a highly conserved family consisting of seven NAD^+^-dependent deacetylases, which share a common 275-amino acid catalytic domain [25], [26]. These seven members are found in distinct subcellular compartments: Sirt1, 6 and 7 are localized to the nucleus, Sirt2 is a cytosolic protein, and Sirt3, 4 and 5 are found in mitochondria [27], [28]. A previous study has shown that Sirt3 knockout mice exhibit striking mitochondrial protein hyperacetylation and no mitochondrial hyperacetylation in mice lacking Sirt4 or Sirt5 [29]. Thus, among these three mitochondrial sirtuins, Sirt3 is the primary deacetylase, which plays an important role in energy production, metabolism, apoptosis and cell signaling [30]–[33]. It has been shown that Sirt3 is enriched in the most metabolically active tissues, such as liver, kidney and heart [34]. Furthermore, Sirt3 facilitates lipid, amino acid and carbohydrate metabolism [35]–[38]. Recent studies have demonstrated that Sirt3 also exhibits a great effect on mitochondrial ROS homeostasis. Sirt3 regulates ROS production by directly binding and deacetylating mitochondrial complex I and II [34], [39]. Sirt3 also regulates ROS clearance via altering the acetylation level of SOD2 [40]–[43]. The mitochondrial oxidation scavenger, SOD2 is thought to play a crucial role in controlling the level of ROS by catalyzing one of the main forms of ROS—superoxide (O_2_^−^) to hydrogen peroxide (H_2_O_2_). SOD2 deficiency is associated with aging and various human diseases [44]. However, whether Sirt3 is involved in the process of central presbycusis, as well as the underlying mechanism of Sirt3, remains unknown.In this study, we determined the generation of lipid peroxidation-MDA in the plasma, the CD level, the ultrastructural changes, and the apoptosis level in the central auditory cortex at different ages of natural aging and D-Gal-treated mimetic aging rats. The level of SOD2 acetylation, SOD2 activity and Sirt3 expression, as well as their possible relationships, were also investigated to determine the mechanism of presbycusis in the central auditory system.

## Materials and Methods

### Ethics statement

All procedures were performed in accordance with the recommendations of the Guide for the Care and Use of Laboratory Animals of the National Institutes of Health. The protocol was approved by the Committee on the Ethics of Animal Experiments of Huazhong University of Science and Technology (Permit Number: S277).

### Animal procedures

Four-week-old Sprague Dawley rats were obtained from the Experimental Animal Center of Tongji Medical College, Huazhong University of Science and Technology. After acclimation for 4 weeks, the 2-month-old rats were randomly divided into two groups: a control group and a D-Gal (Sigma Chemical, St. Louis, MO) group. The rats in the D-Gal group were injected subcutaneously with D-Gal (500 mg/kg/d) for 8 weeks; the rats in the control group were injected with the same volume of vehicle (0.9% saline) on the same schedule. After the last injection, both the NS and D-Gal groups were divided into 3 age subgroups: 4-month-old (just after the injection), 10-month-old (6 months after the last injection), and 18-month-old (14 months after the last injection). All rats were housed in an air-conditioned animal facility at 23°C±2°C, with 50%–60% relative humidity, under a 12 h light/dark cycle and were fed with standard rodent chow and water.

### Measurements of SOD2 activity and MDA level

The SOD2 activity and MDA level in the auditory cortex were quantified using colorimetric kits (Jiancheng, Nanjing, China) according to the manufacturer's instructions[24].

### Transmission electron microscopy (TEM)

The rats (n = 6 per subgroup) were anesthetized with a combination of ketamine (100 mg/kg) and chlorpromazine (5 mg/kg) via intraperitoneal injection and then perfused transcardially with a brief wash of 0.9% oxygenated saline, which was followed by 3% glutaraldehyde in 0.1 M phosphate buffer (pH 7.4). Following perfusion, the brain was dissected from the skull, and the auditory cortex was separated and immersed overnight in the same fixative. After postfixation in 1% osmium tetroxide for 2 h at room temperature, the tissues were dehydrated in a graded ethanol series and then treated with propylene oxide for approximately 30 min prior to embedding in a graded araldite mixture for block preparation. Serial ultrathin sections were distributed on copper grids and stained with uranyl acetate, followed by lead citrate. The ultrastructure of the stained sections was examined using a Transmission Electron Microscope (FEI TecnaiG^2^12, Phillips, Holland). Three pieces of ultrathin sections from each sample were selected for ultrastructural study using TEM.

### DNA extraction and cDNA generation

After the last injection (just after the injection was completed, 6 months after the last injection, and 14 months after the last injection), 6 rats from each subgroup were sacrificed and both sides of the auditory cortex were rapidly removed. The samples were stored at −80°C until further processing. Total DNA was extracted from 20 mg of tissue using the Genomic DNA Purification Kit (Tiangen Biotech Co., LTD, Beijing, China) according to the manufacturer's instructions. The DNA concentration of each sample was measured using the Gene Quant Pro DNA/RNA Calculator (BioChrom, Cambridge, UK). Total tissue RNA was extracted using Trizol reagent (Invitrogen Inc, Carlsbad, CA) according to the manufacturer's protocol. cDNA was reverse transcribed using the PrimeScript RT reagent Kit (TaKaRa, Dalian, China). The RNA and cDNA of each sample were then analyzed using the Gene Quant Pro DNA/RNA Calculator to assess the concentrations and purification. The cDNA samples were stored at −20°C until further use.

### Quantification of mtDNA 4834 bp deletion

The proportion of mtDNA 4834 bp deletion was determined using the TaqMan real-time PCR assay. The D-loop region copy number was used as a measurement of the total amount of mtDNA in a given tissue sample. The primers and probes for the D-loop region and mtDNA 4834 bp deletion have been previously described by Nicklas et al. [21]. PCR amplification was performed using the StepOnePlus™ Real-Time PCR System (Applied Biosystems, Foster City, CA) in a 20 µl reaction volume consisting of 10 µl of a 2×TaqMan PCR mix (TaKaRa, Dalian, China), 0.4 µl of a 50×ROX reference dye, 0.4 µl of each forward and reverse primer (10 µM), 0.2 µl of each probe (10 µM), 4 µl of the sample DNA (10 ng/µl), and 4.6 µl of distilled water. The cycling conditions include an initial phase at 95°C for 30 s, then 40 cycles at 95°C for 5 s and at 60°C for 30 s. The abundance of mtDNA was calculated as the measurement of the cycle threshold (CT), which is the PCR cycle number at which the fluorescence measurement reaches a set value. The difference in CT values was used as the measurement of relative abundance; ΔCT (CT_deletion_ - CT_D-loop_) was used to calculate the abundance of the mtDNA 4834 bp deletion. The relative expression (RE) indicates the factorial difference in the deletions between the experimental group and control group. The RE was calculated as 2^−ΔΔCT^, where ΔΔCT = ΔCT_mtDNA deletion in experimental group_−ΔCT_mtDNA deletion in control group._

### Gene expression analysis using real-time PCR

Quantitative real-time PCR was performed by applying real-time SYBR Green PCR technology with the use of the 7300 Real-Time PCR System (Applied Biosystems, Foster City, CA). Validated primers were designed for each target mRNA. The primer pairs used for Sirt3, SOD2 and β-actin were as follows: Sirt3 forward, 5*′*-TGCACGGTCTGTCGAAGGTC-3′; Sirt3 reverse, *5′*-A TGTCAGGTTTCACAACGCCAGTA-3′*;* SOD2 forward, 5′-G AGCAAGGTCGCTTACAGA-3′; SOD2 reverse, 5′-C TCCCCAGTTGATTACATTC-3′; β-actin forward, 5′-CCT GGAGAAGAGCTATGAGC-3′; β-actin reverse, 5′-A CAGGATTCCATACCCAGG-3′. The amplification conditions were as follows: 30 s at 95°C, and then 40 cycles of 5 s at 95°C, 30 s at 60°C, and 35 s at 72°C. An internal standard was used to normalize the relative gene expression levels. The reaction specificities were verified using melting curve analysis. The relative expression was calculated from the differences in the CT values between the target mRNA and an internal standard (β-actin). The change in the relative mRNA levels between the experimental group and control group was analyzed using the 2^-ΔΔct^ method.

### Western blotting analyses

The protein expression levels of Sirt3 and SOD2 in the auditory cortex were analyzed using Western blotting analyses. Thirty-six rats (n = 6 per subgroup) were sacrificed, and the auditory cortical tissues were dissected. The total protein was extracted using RIPA Lysis Buffer (Beyotime, Haimen, China) according to the manufacturer's instructions. Next, 100 µl of fresh supernatant from each EP tube was transferred to a new EP tube for immunoprecipitation. Protein concentrations were determined using the BCA Protein Assay Kit (Beyotime, Haimen, China). Twenty micrograms of each protein lysate was separated using 12% SDS-polyacrylamide gels and transferred onto a PVDF membrane. The blots were blocked with 5% non-fat milk in TBS and incubated overnight at 4°C with the appropriate dilution of primary antibodies: anti-Sirt3 (diluted 1∶1000, Cell Signaling Technology) and anti-SOD2 (diluted 1∶500, Santa Cruz). After washing the membranes to remove excess primary antibody, the membranes were incubated for 1 h at room temperature with the appropriate secondary antibodies at a dilution of 1∶3000-1∶5000. The membranes were washed 3 times and then visualized using ECL Plus (Beyotime, Haimen, China). GAPDH was used as an internal control. Middle-aged and aged rats were processed according to the same schedule.

### Immunoprecipitation

Sufficient amounts of anti-SOD2 or anti-Sirt3 antibody (Santa Cruz) were added to the fresh protein supernatant and incubated at 4°C overnight. Next, 25 µl of protein A+G agarose beads (Beyotime, Haimen, China) was added and incubated for 3 h at 4°C. The mixture was then centrifuged at 2500× *g* for 5 min at 4°C°C, and the supernatant was discarded. The precipitate was washed five times with ice-cold PBS. After washing, the immunocomplex was boiled in 2x SDS buffer for 5 min. The supernatant was then collected by centrifugation and subjected to western blotting analyses with anti-acetyl-lysine antibody (1∶1000, Cell Signaling Technology), anti-SOD1(1∶500, Santa Cruz), anti-SOD2 (1∶500, Santa Cruz), anti-SOD3 (1∶500, Santa Cruz), anti-Sirt3 (1∶500, Santa Cruz), anti-Sirt4 (1∶500, Santa Cruz), and anti-Sirt5 antibodies (1∶500, Santa Cruz). The control for these IP experiments was normalized against rabbit IgG.

### Immunofluorescence

Using an immunofluorescence method and a laser-scanning confocal microscope (Nikon, Japan), we detected the distribution of Sirt3 in the auditory cortex. Thirty-six rats (n = 6 per subgroup) were transcardially perfused with saline followed by 4% paraformaldehyde solution. The brains were removed and postfixed in 4% paraformaldehyde overnight at 4°C. A 5-µm section was deparaffinized in xylene and rehydrated through graded concentrations of ethanol. Following antigen retrieval, nonspecific binding was blocked with donkey serum albumin for 1 h at room temperature. Next, the anti-Sirt3 antibody (diluted 1∶50, Santa Cruz) was added and incubated overnight at 4°C. After three washes with PBS, the sections were incubated for 1 h with fluorescently tagged secondary antibody. Control staining was performed in the absence of primary antibody.

### TUNEL staining

Thirty-six rats (n = 6 per subgroup) were used for TUNEL staining. Serial 5-µm coronal sections containing the auditory cortex were detected using the In Situ Cell Death Detection Kit (Roche Diagnostics, Mannheim, Germany) according to the manufacturer's instructions. Apoptotic nuclei were visualized using the peroxidase-DAB reaction and counterstained with hematoxylin [15]. The percentage of TUNEL-positive cells was quantified as follows: TUNEL-positive cells (%)  = 100× (apoptotic cells/total cells) [45].

### Statistical analysis

All of the results are representative of at least three independent experiments for each group. The data are presented as the mean ± SEM. The analysis was performed using SPSS 13.0 software. The statistical significance was determined using one-way ANOVA. Differences with a p-value<0.05 were considered statistically significant.

## Results

### Increased MDA levels and decreased SOD2 activity in natural aging and D-Gal-induced aging rats

**Figure 1:**
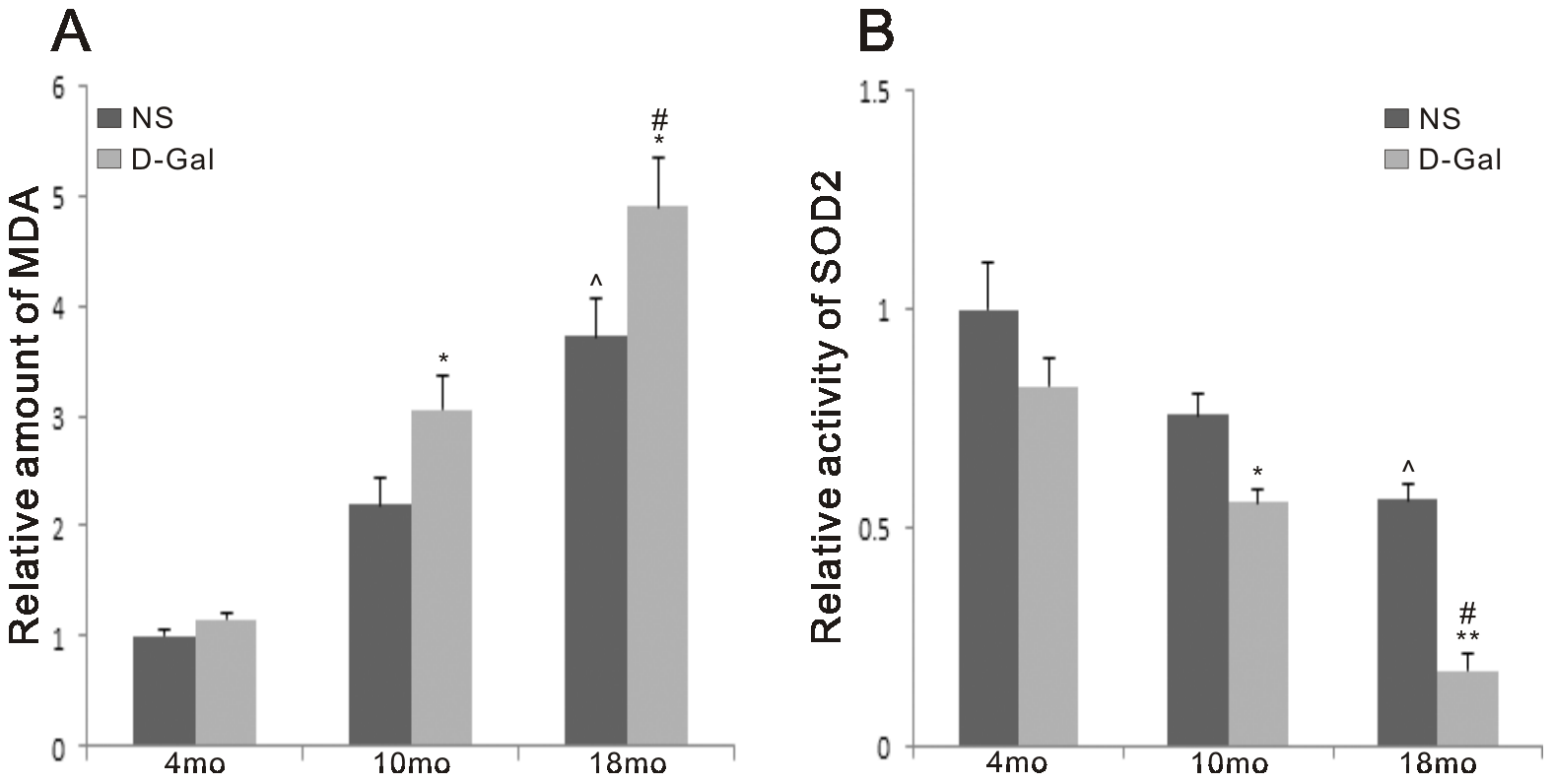
MDA levels and SOD2 activity in the auditory cortex. Effects of age and D-Gal on MDA levels and SOD2 activity in the auditory cortex. The level of MDA was increased in the D-Gal groups, while the activity of SOD2 was decreased in the D-Gal groups. *Significantly different from the NS groups (*P<0.05, **P<0.01). ^∧^Significantly different from the 4-month-old NS group (P<0.01). #Significantly different from the 4-month-old D-Gal group (P<0.01).

Oxidative stress plays a central role in the pathogenesis of AHL [46], [47]. To understand the pathway by which age and D-Gal affect central presbycusis, the SOD2 activity and MDA levels in the auditory cortex were determined. As shown in Fig. 1, we found that the generation of MDA was significantly elevated in the D-Gal groups, while the activity of SOD2 showed a significant decrease. Compared to the NS groups, the MDA levels of the 4-, 10- and 18-month-old rats in the D-Gal group were increased by 1.18-, 1.39- (P<0.05) and 1.32-fold (P<0.05), respectively. In the NS or D-Gal group, the MDA level showed a 3.72- or 4.22-fold (P<0.01) increase between 4- and 18-month-old rats. However, compared to the NS group, the activity of SOD2 in the age-matched D-Gal group was reduced by 1.21-, 1.36- (P<0.05) and 4.50-fold (P<0.01), respectively. In comparison with the 4-month-old NS or D-Gal group, the activity of SOD2 in the 18-month-old NS and D-Gal group was decreased by 1.77- (P<0.01) and 6.58-fold (P<0.01), respectively. SOD2 was the primary scavenger of superoxide, and its activity was decreased in central presbycusis.

### Increase of the CD levels in natural aging and D-Gal-induced aging rats

**Figure 2:**
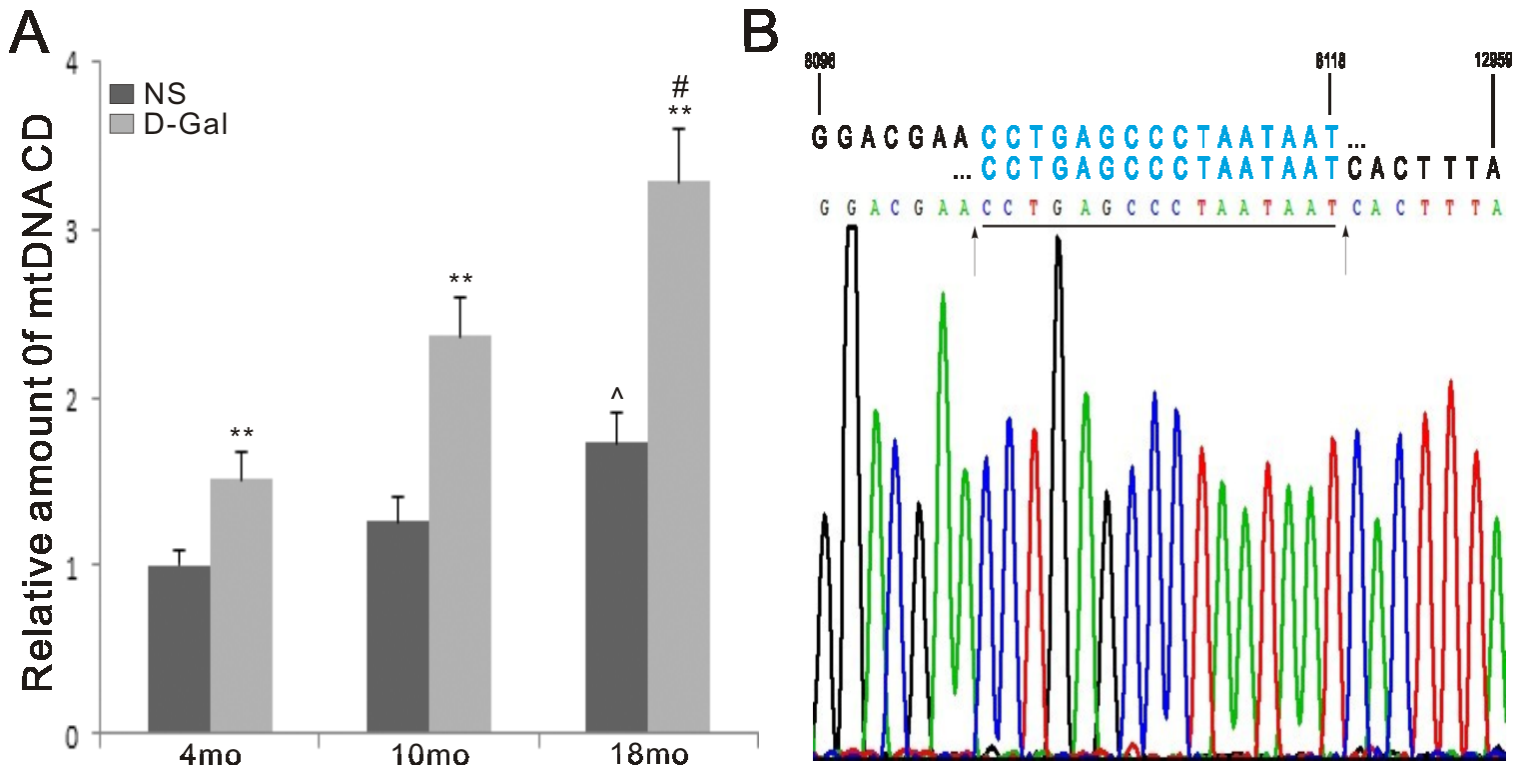
mtDNA common deletion in the auditory cortex and schematic diagram. A. Accumulation of mtDNA common deletion was measured using quantitative PCR. The levels of the mtDNA common deletion were significantly increased in the D-Gal groups compared to the corresponding NS groups. A significant difference was also found between the 4- and 18-month-old NS or D-Gal group. **Significantly different from the NS groups (P<0.01). ^∧^Significantly different from the 4-month-old NS group (P<0.01). # Significantly different from the 4-month-old D-Gal group (P<0.01). Data were expressed as the mean ± SEM. (n = 6 per subgroup). B. Schematic diagram and sequences of the 4834 bp mtDNA deletion (nt 8103 – nt 12936 or nt 8119 – nt 12952). Bold black letters indicate the nucleotide sequences flanking the breakpoints of the deleted mtDNA, and the bold blue letters indicate the direct repeats. Sequences of the PCR products are shown below the in schematic diagram. The arrowheads indicate the potential breakpoints.

Mitochondria are the primary sites of ROS generation, and are the most vulnerable to ROS attack. mtDNA mutations are inevitable during aging. To evaluate the accumulation of CD in the auditory cortex, a quantitative PCR (TaqMan probe) assay was performed. Dual-labeled fluorescent DNA probes specific for the new fusion sequence, which was present only in mutant mtDNA harboring the 4834 bp deletion, were generated. As observed in Fig. 2, the levels of the CD were significantly increased in the D-Gal groups compared with the age-matched NS groups. We further found a significant difference between the 4- and 18-month-old rats in both groups. Compared with the NS group, the accumulation of the CD in the age-matched D-Gal group was increased by 1.51-, 1.87- and 1.90-fold (P<0.01), respectively. In the NS groups, the level of the CD was increased by 1.73-fold (P<0.01) between the 4- and 18-month-old rats. In the D-Gal groups, the level of the CD was increased by 2.17-fold (P<0.01) between the 4- and 18-month-old rats. This result may indicate that the CD is a biomarker for aging.

### Age-related changes in the ultrastructural morphology in the auditory cortex

**Figure 3:**
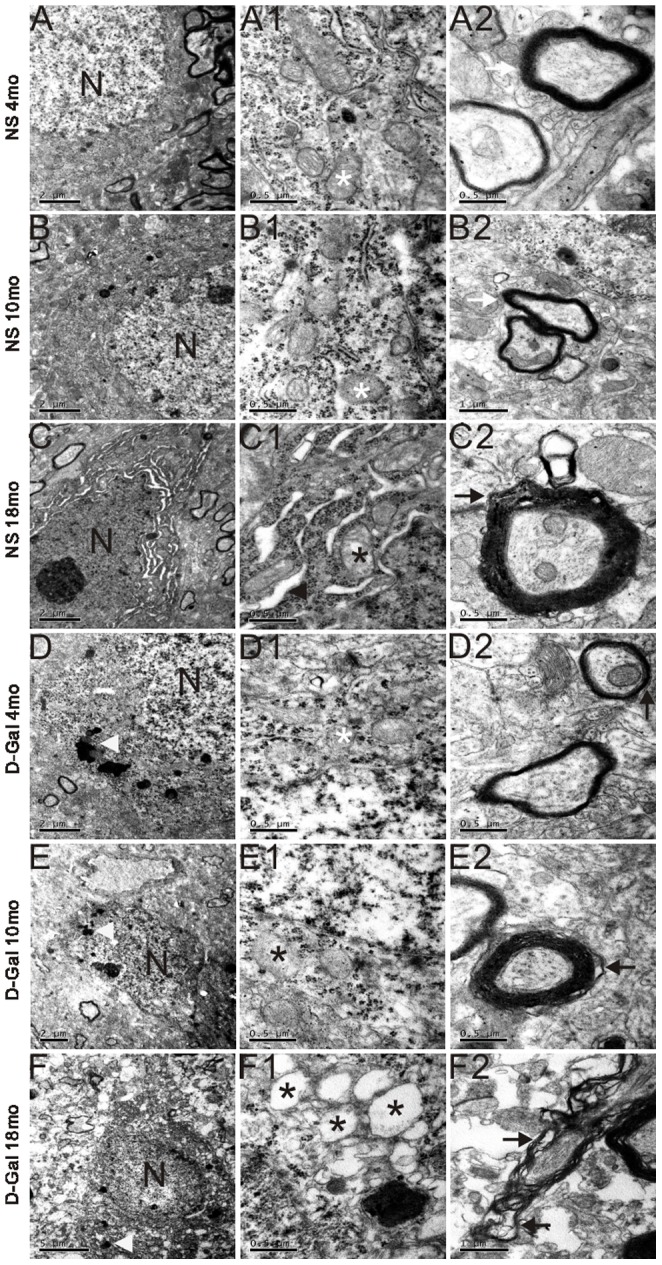
Age-related changes in the ultrastructural morphology in the auditory cortex. Ultrastructural changes in the auditory cortex at different ages in the NS and D-Gal groups. Normal nucleus, uniformly chromatin, and normal mitochondria (white asterisks) are shown in the 4- and 10-month-old NS group and the 4-month-old D-Gal group, irregular nucleus, condensed chromatin, and swollen and vacuolated mitochondria (black asterisks) were found in the 18-month-old NS group, 10- and 18-month-old D-Gal group. Intact and compact myelin (white arrows) was in the 4- and 10-month-old NS group, swollen and disrupted myelin (black arrows) was in the 18-month-old NS group and different D-Gal groups. Enlarged endoplasmic reticulum was found in the 18-month-old NS group. Accumulated lipofuscin (white arrowheads) were shown in the D-Gal groups.

In the NS groups, neurons of the auditory cortex displayed no obvious ultrastructural changes in 4- and 10-month-old rats. Intact nuclear membrane and uniform chromatin can be observed in Fig. 3A and B. In addition, abundant mitochondria and rough endoplasmic reticulum were present in the cytoplasm (Fig. 3A1 and B1). The myelin sheaths of nerve fibers were also intact and compact (Fig. 3A2 and B2). However, in the 18-month-old rats, enlarged endoplasmic reticulum, swollen mitochondria and disrupted myelin were observed (Fig. 3C, C1 and C2). In the D-Gal groups, neurons of the auditory cortex displayed neurodegeneration with age. The nuclear morphology was irregular, the chromatin was condensed, there was an accumulation of lipofuscin, the mitochondria were swollen, and the myelin was disrupted in the 10-month-old D-Gal rats (Fig. 3E, E1 and E2). Moreover, these changes were more significant in the 18-month-old D-Gal rats (Fig. 3F, F1 and F2).

### Increased cell apoptosis in D-Gal-induced aging rats

**Figure 4:**
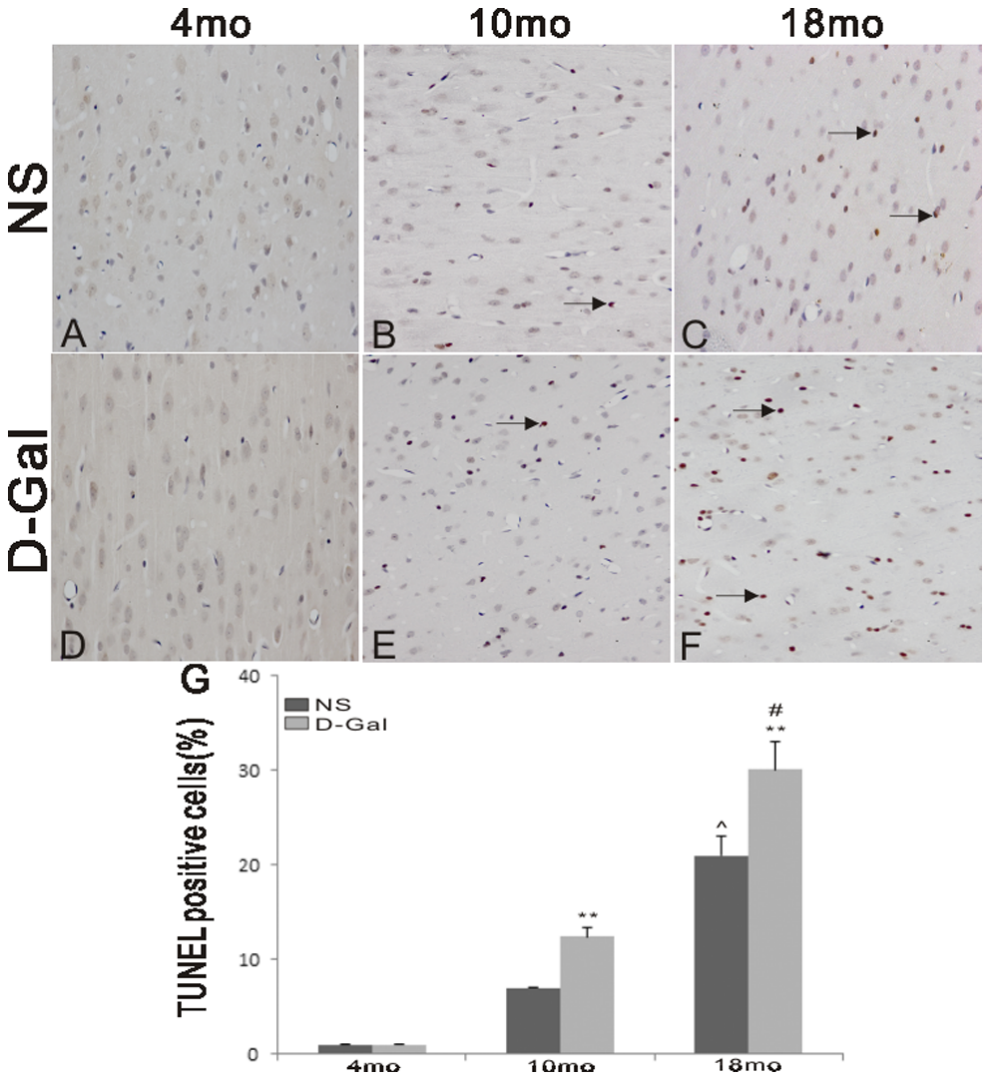
Cell apoptosis in the auditory cortex. Apoptotic cells were determined using TUNEL staining. No TUNEL-positive cellswere observed in the 4-month-old NS and D-Gal groups. The number of TUNEL-positive cells in the 10- and 18-month-old D-Gal group was significantly increased compared to the age-matched NS groups. **Significantly different from the NS groups (P<0.01). ^∧^Significantly different from the 4-month-old NS group (P<0.01). # Significantly different from the 4-month-old D-Gal group (P<0.01). Magnification: 400×. (n = 6 per subgroup).

Oxidative stress results in mtDNA damage, functional decline and cell apoptosis. To investigate the effects of D-Gal and age on cell apoptosis in the auditory cortex, TUNEL staining was performed. No TUNEL-positive cells were found in the 4-month-old NS and D-Gal groups (Fig. 4A and D). However, the number of TUNEL-positive cells was significantly increased in the 10- and 18-month-old D-Gal groups compared with the age-matched NS groups (P<0.01).

### Decreased mRNA expression of Sirt3 and SOD2 in natural aging and D-Gal-induced aging rats

**Figure 5:**
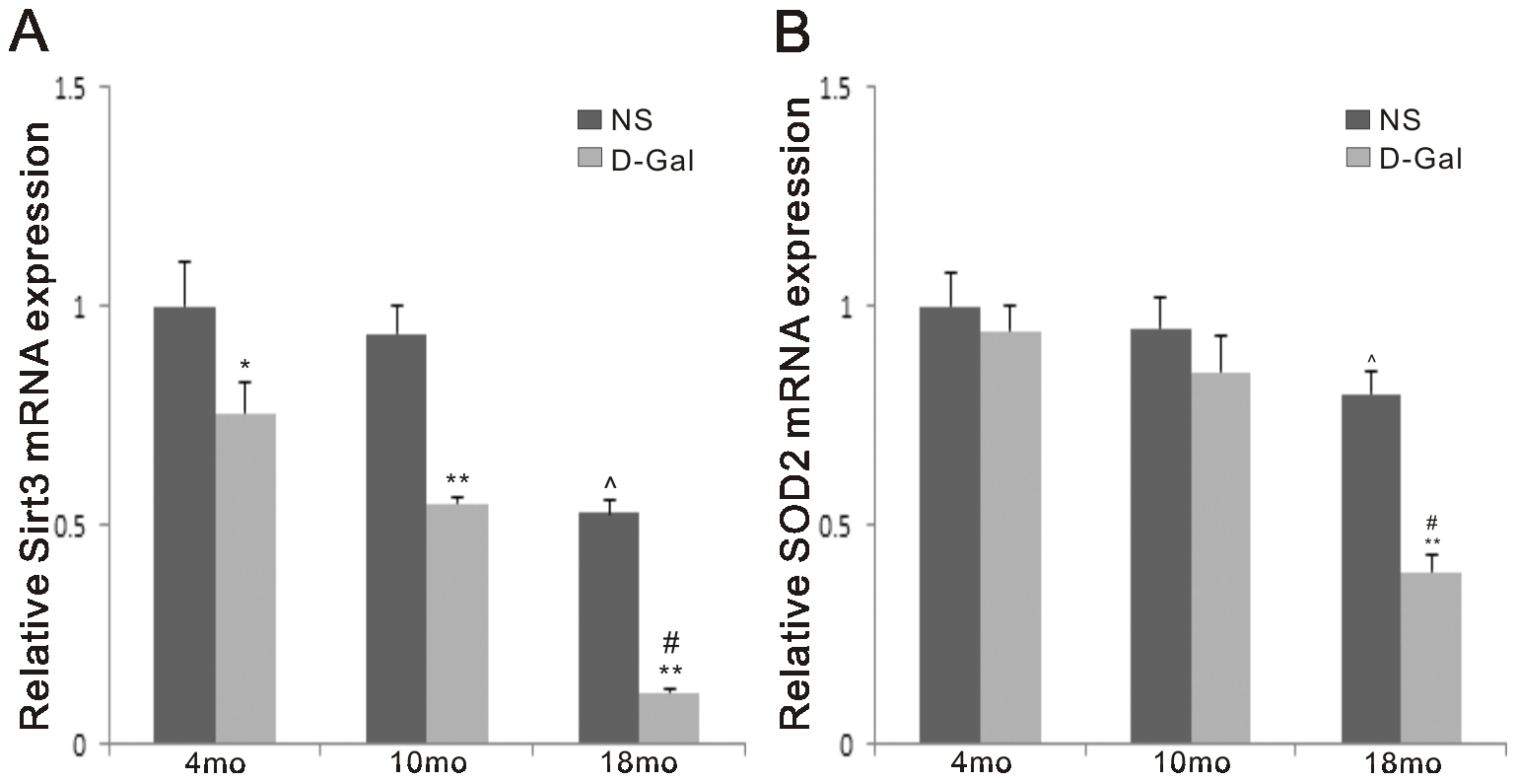
mRNA expression of Sirt3 and SOD2 in the auditory cortex. Quantitative RT-PCR was used to measure the effect of age and D-Gal on Sirt3 and SOD2 mRNA expression. The levels of Sirt3 mRNA expression in the three D-Gal groups were significantly lower than the NS groups, but a significant decrease of SOD2 mRNA expression was only found between the 18-month-old NS and D-Gal groups. Both gene expressions were significantly decreased with age. *Significantly different from the NS groups (*P<0.05, **P<0.01). ^∧^Significantly different from the 4-month-old NS group (P<0.05). # Significantly different from the 4-month-old D-Gal group (P<0.01). Data are expressed as the mean ± SEM. (n = 6 per subgroup)

Studies have shown that Sirt3 deacetylates the critical antioxidant enzyme SOD2 in the mitochondrial matrix [40], [42]. Deacetylation of SOD2 results in increased antioxidant activity and enhanced scavenging of ROS. To determine the effect of D-Gal and age on the mRNA levels of Sirt3 and SOD2, quantitative RT-PCR assays were performed. Compared to the corresponding NS groups, the levels of Sirt3 and SOD2 mRNA expression were decreased in D-Gal groups (Fig. 5A and B). Furthermore, a comparison of the 4-, 10- and 18-month-old NS group revealed that Sirt3 mRNA expression in the age-matched D-Gal group was reduced by 1.32- (P<0.05), 1.70- (P<0.01) and 4.46-fold (P<0.01), and the SOD2 expression was reduced by 1.06-, 1.11- (P>0.05) and 2.04-fold (P<0.01), respectively. Furthermore, we found that the expression of Sirt3 and SOD2 changed with age; however, age had less of an effect on SOD2 mRNA expression. Compared to the 4-month-old NS, Sirt3 and SOD2 expression in 18-month-old NS group was decreased by 1.90- (P<0.01) and 1.25-fold (P<0.05), respectively. Compared to the 4-month-old D-Gal group, Sirt3 and SOD2 expression in the 18-month-old D-Gal group was decreased by 4.41- and 2.41-fold (P<0.01), respectively.

### SOD2 and Sirt3 physically interact in the auditory cortex

**Figure 6:**
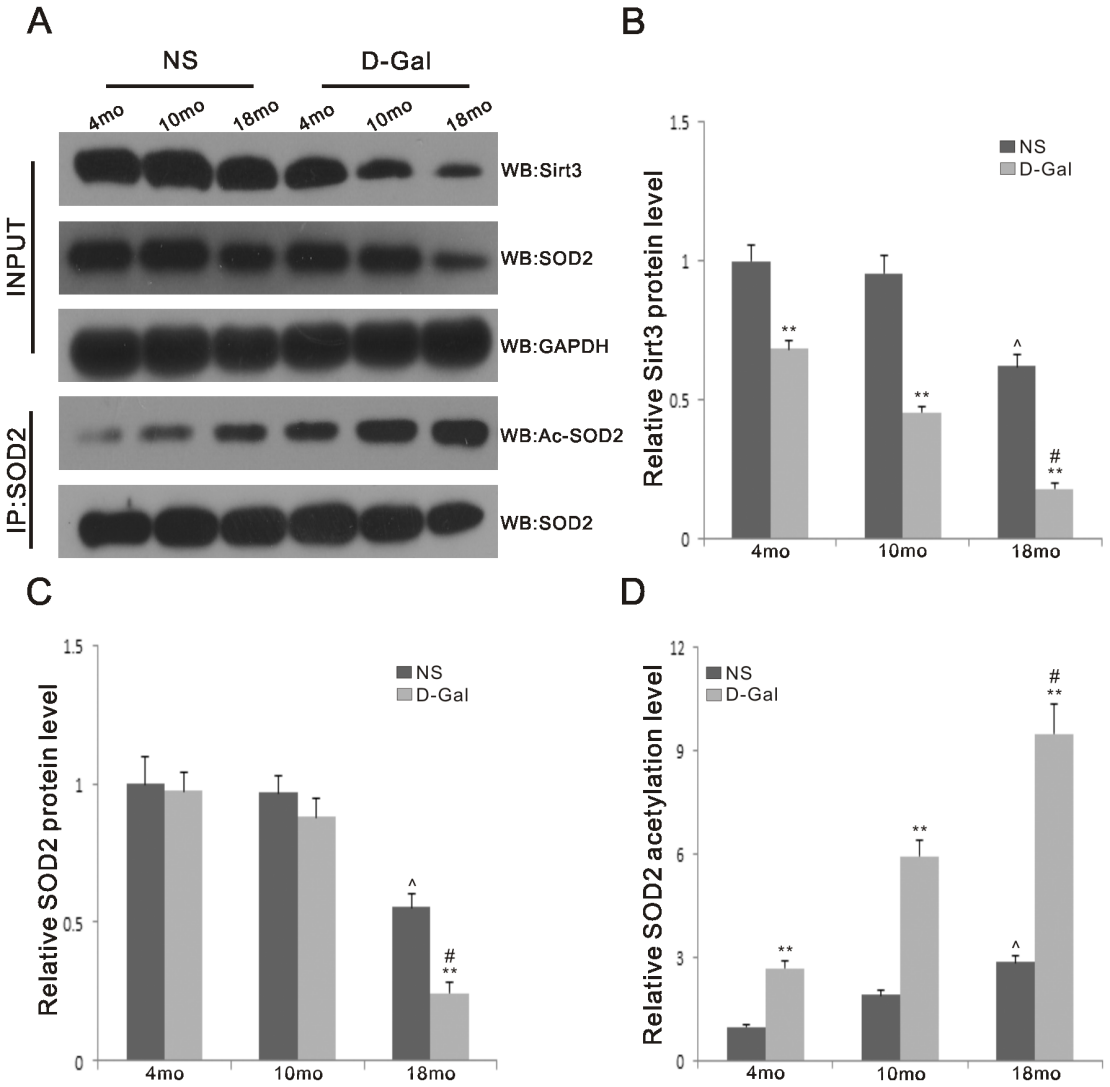
Physical interaction between SOD2 and Sirt3 in the auditory cortex. A. Endogenous SOD2 was immunopurified from the auditory cortex with anti-SOD2 antibody, followed by western blotting with anti-SIRT3, anti-Sirt4 and anti-Sirt5 antibodies. B. Endogenous Sirt3 was immunopurified from the auditory cortex with anti-Sirt3 antibody, followed by western blotting with anti-SOD2, anti-SOD1 and anti-SOD3 antibodies.

Given that SOD2 is a mitochondrial oxidation scavenger, we determined the interaction between SOD2 and the three mitochondrially localized sirtuins-Sirt3, Sirt4 and Sirt5. In addition, we determined the interaction between Sirt3 and the SOD family members SOD1, SOD2 and SOD3. Immunoprecipitation and western blotting analyses demonstrated that Sirt3, but not the other sirtuins tested, binds to SOD2 (Fig. 6A). Moreover, SOD2, but not other SOD family members tested, binds to Sirt3 (Fig. 6B). Our results are supported by those of a previous study [41].

### Decreased protein levels of Sirt3 and SOD2 and increased acetylation levels of SOD2 in natural aging and D-Gal-induced aging rats

**Figure 7:**
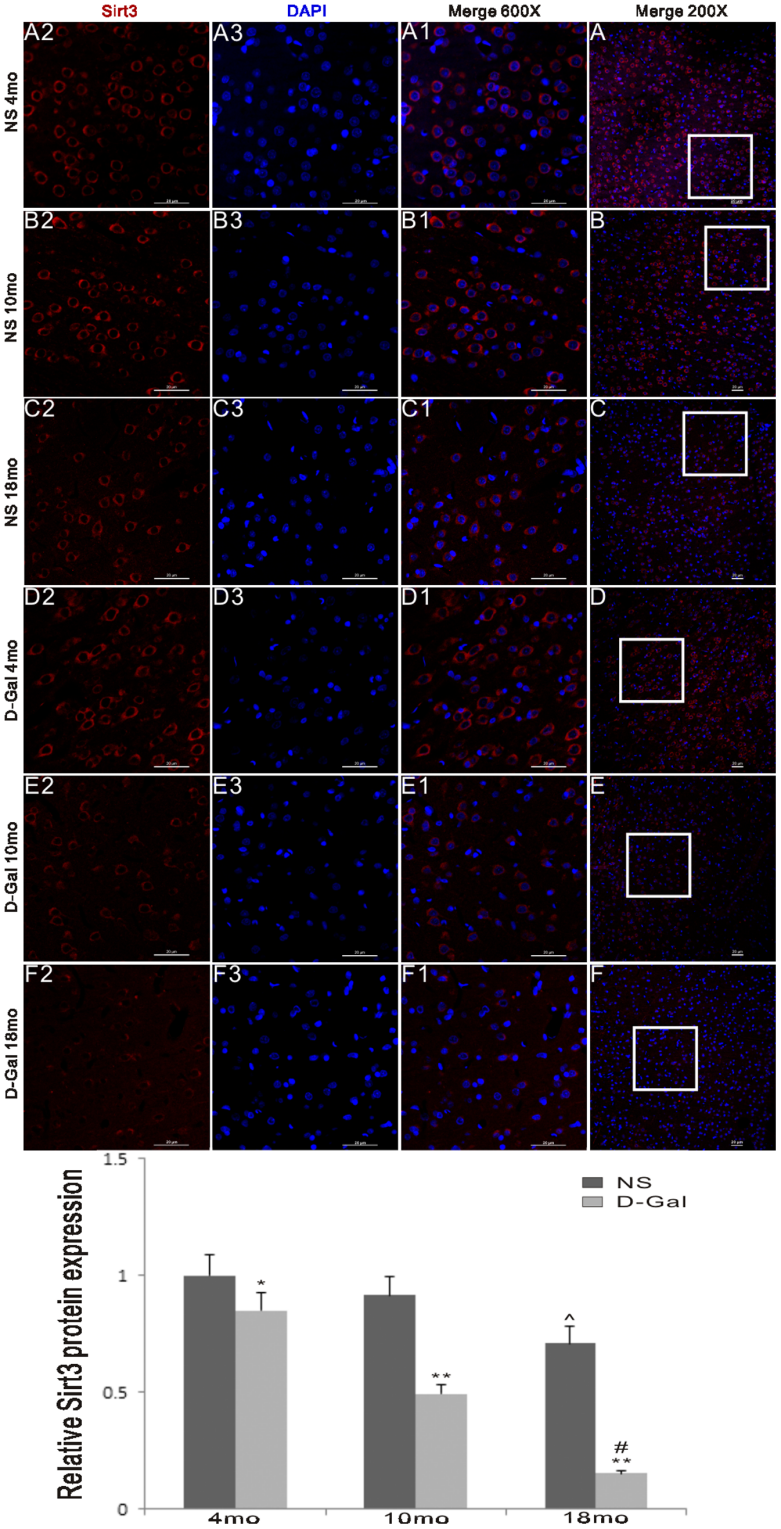
Protein levels of Sirt3 and SOD2 and acetylation levels of SOD2 in the auditory cortex. A. Top panels: Western blotting analysis of Sirt3 and SOD2 in the auditory cortex from the 4-, 10- and 18-month-old rats in the NS and D-Gal groups. GAPDH was used as a reference. Lower panels: Endogenous acetylated SOD2 was isolated by immunoprecipitation with anti-SOD2 antibody followed by western blotting with anti-acetyl-lysine antibody. SOD2 was used as a reference. (n = 6 per subgroup) B. Quantification of the amounts of total Sirt3 protein (Fig. 7B) from (Fig. 7A).The levels of Sirt3 protein were significantly decreased in the D-Gal groups compared to the NS groups, as well as in the 18-month-old groups compared to the 4-month-old groups. C. Quantification of the amounts of total SOD2 protein (Fig. 7C) from (Fig. 7A). The levels of SOD2 protein was significantly decreased between the 18-month-old D-Gal and NS groups. Significant differences were also found between the 4- and 18-month-old groups. D. Quantification of the amounts of SOD2 acetylation (Fig. 7D) from (Fig. 7A). The levels of SOD2 acetylation were significantly increased in the D-Gal groups compared to the NS groups, as well as in the 18-month-old groups compared to the 4-month-old groups. **Significantly different from the NS groups (P<0.01). ^∧^Significantly different from the 4-month-old NS group (P<0.01). #Significantly different from the 4-month-old D-Gal group (P<0.01).

Sirt3 is a mitochondrial NAD^+^-dependent deacetylase responsible for SOD2 deacetylation. SOD2 activity is tightly regulated by acetylation on its lysine residues [40], [42]. We further measured Sirt3 and SOD2 protein levels using Western blotting analyses and SOD2 acetylation levels using SOD2 immunoprecipitation followed by Western blotting using an anti-acetyl-lysine antibody. Our results showed that compared to the NS group, the protein levels of Sirt3 in the age-matched D-Gal group were reduced by 1.46-, 2.10- and 3.47-fold (P<0.01), and those of SOD2 were reduced by 1.03-, 1.10- (P>0.05) and 2.27-fold (P<0.05), respectively. Both Sirt3 and SOD2 protein expression decreased with age. Compared to the 4-month-old NS group, the levels of Sirt3 and SOD2 in the 18-month-old NS group were decreased by 1.60- and 1.79-fold (P<0.01). Compared to the 4-month-old D-Gal group, the levels of Sirt3 and SOD2 in the 18-month-old D-Gal group were decreased by 3.82- and 3.98-fold (P<0.01), respectively. As shown in Fig. 7D, we found that, in comparison with the corresponding NS group, the acetylation level of SOD2 in the 4-, 10- and 18-month-old D-Gal group was increased by 2.70-, 3.09- and 3.31-fold (P<0.01), respectively. We also found that the acetylation levels of SOD2 were significantly higher in 18-month-old rats compared to 4-month-old rats in both the NS and D-Gal groups. Compared to the 4-month-old NS or D-Gal group, the acetylation level of SOD2 in the 18-month-old NS or D-Gal group was increased by 2.87- or 3.52-fold (P<0.01), respectively. As shown in Fig. 1B and Fig. 7D, we determined that the activity of SOD2 was inversely proportional to the acetylation level. Therefore, when the acetylation level was higher, the activity of SOD2 was lower.

### Decreased Sirt3 protein expression in natural aging and D-Gal-induced aging rats

**Figure 8:**
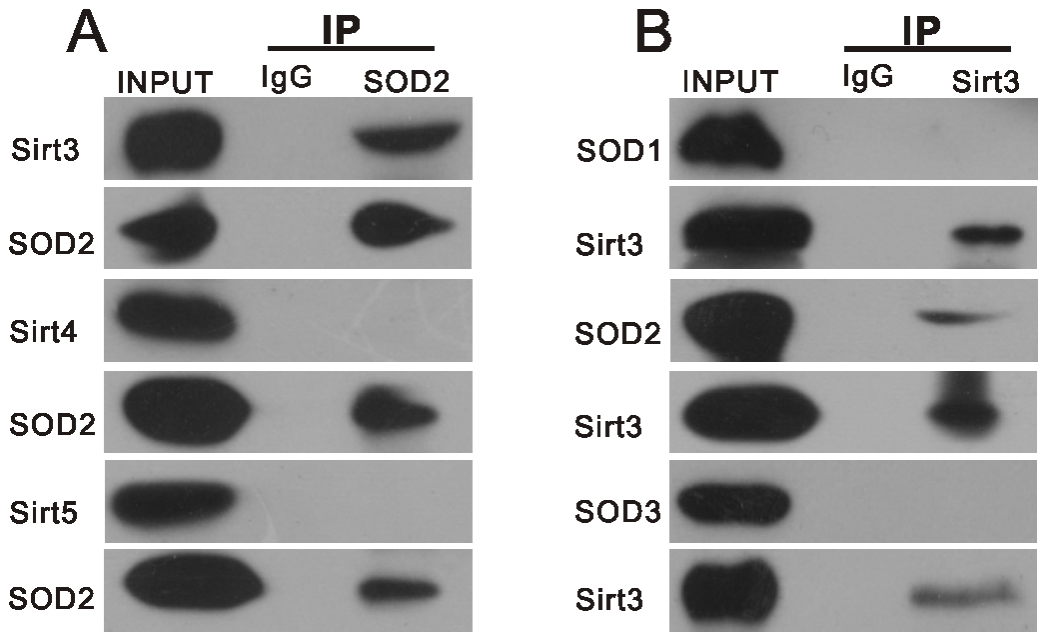
Sirt3 protein expression in the auditory cortex. An immunofluorescence assay was used to measure the effects of age and D-Gal on Sirt3 protein expression in the auditory cortex. The levels of Sirt3 protein expression in the D-Gal groups were significantly lower compared to the NS groups. The levels were also decreased in the 18-month-old groups compared to the 4-month-old groups.

To further characterize the age-related changes in the protein expression of Sirt3 in the auditory cortex, we performed an immunofluorescence assay. According to a previous study, Sirt3 is expressed in neurons and glia throughout the central nervous system [48]. Our results showed that Sirt3 protein expression in the auditory cortex of the D-Gal group was significantly decreased compared to the age-matched NS group. Moreover, the levels of Sirt3 protein were significantly lower in the 18-month-old animals compared to the 4-month-old controls (Fig. 8).

## Discussion

It is well established that the accumulation of ROS originating from the mitochondrial electron transport chain results in aging and age-associated diseases [4], [49], [50]. D-Gal can be converted into galactitol, which cannot be metabolized and will accumulate in the cell, by aldose reductase, resulting in osmotic stress and excessive ROS production [51]. Long-term administration of D-Gal induces changes that resemble natural aging in animals, such as a shortened lifespan [52], [53], cognitive dysfunction [54], neurodegeneration [55], oxidative stress [56], decreased immune responses [57], and advanced glycation endproduct (AGE) formation [58]. Thus, chronic systemic exposure of rodents to D-Gal causes an acceleration of senescence and has been used as a mimetic aging model. AHL consists of two types of deficits; one type of deficit is peripheral in origin, and the other is centrally mediated. Age-related dysfunction of the central auditory cortex reduces the ability to localize sounds, which is a critical component for segregating different acoustic objects and events [59]. However, poor understanding of the explicit pathogenesis of central presbycusis is less helpful in treating this disease. As a result, the mechanisms of AHL concerning the central auditory cortex have been investigated using D-Gal-induced mimetic aging rats.

Previous studies have indicated that oxidative stress plays a causal role in AHL [19]–[22]. Our present data further showed that accumulated oxidative stress is involved in central presbycusis because the MDA levels in the auditory cortex of the D-Gal groups at different ages were significantly higher than those of age-matched NS groups, while the SOD2 activity was remarkably lower. Oxidative stress can cause oxidative damage to biomolecules, such as proteins, lipids and DNA. Because ROS are generated mainly as by-products of mitochondria respiration, it appears evident that mtDNA is vulnerable to attack by oxidative damage. The most frequent and best-characterized age-related mtDNA mutation is the CD. We further found that the CD was significantly accumulated in the auditory cortex of mimetic aging rats compared with controls. CD accumulation was also found in the auditory cortex of 18-month-old rats. In addition, our findings support the idea that the CD is a biomarker for AHL. Moreover, we observed age-related changes in the ultrastructural morphology of the auditory cortex from the NS and D-Gal groups. Irregular nuclei, condensed chromatin, swollen mitochondria, and disrupted myelin could be found in the 18-month-old NS group and 10-month-old D-Gal group. Furthermore, these degenerative changes were more serious in the 18-month-old D-Gal group. In addition, increased cell apoptosis was observed in the natural aging and D-Gal-induced animals. Our studies provide more convincing evidence for the toxicity of accumulated ROS in the central auditory cortex because oxidative stress causes damage to mtDNA, resulting in an increase in CD rates and decline in mitochondrial function, leading to ultrastructural degenerative changes and cell apoptosis. These events subsequently lead to the progression of central presbycusis. While oxidative stress is involved in central presbycusis, the molecular mechanisms by which ROS accumulates remain unknown. Because 90% of cellular ROS are produced in mitochondria, it is logical to investigate ROS suppressors in mitochondria. Thus, SOD2, the major mitochondrial antioxidant scavenger, was investigated in the present study. In mitochondria, SOD2 converts superoxide generated by the respiratory chain into hydrogen peroxide, and protects mitochondria from harmful ROS attack. It is well know that SOD2 is regulated via transcriptional activation. Recently, acetylome proteomic studies have shown that SOD2 is an acetylated protein, indicating a new regulation of SOD2 at the post-translational level. In addition, the antioxidant activity of SOD2 can be regulated by acetylation, and the activity of SOD2 is inversely proportional to the acetylation level [41], [42]. Therefore, when the acetylation level is higher, the activity of SOD2 is lower. The results of our study of the auditory cortex are supported by earlier findings. The acetylation levels of SOD2 were significantly elevated in the D-Gal groups compared with the age-matched NS groups, but the SOD2 activity was significantly decreased. Moreover, Sirt3, a mitochondrial matrix protein, is a key regulator of mitochondrial protein acetylation status [60]. Previous studies have shown that Sirt3 mRNA expression is 70% lower in hematopoietic stem/progenitor cells (HSPCs) of old mice compared to those of young mice, and upregulation of Sirt3 could rescue the functional defects of aged HSCs [61], [62]. Decreased Sirt3 expression is also found in human cells harboring the 4977 bp mtDNA deletion [63]. In addition, Sirt3 knockout mice exhibit increased oxidative stress in skeletal muscle and accelerated aging during fasting [62], [64]. Sirt3 deacetylates SOD2 in response to calorie restriction or ionizing radiation or ROS stress, indicating that SOD2 is a major downstream mediator of Sirt3 in reducing cellular ROS [40]-[42]. However, whether Sirt3 is involved in central presbycusis remains unclear. In this study, an immunofluorescence assay showed that the expression of Sirt3 in the auditory cortex was significantly decreased in the D-Gal groups compared with the aged-matched NS groups. We further tested the effect of D-Gal and age on Sirt3 mRNA expression and found similar results. Moreover, Western blotting analyses and immunoprecipitation assays were performed to investigate whether Sirt3 is physically associated with SOD2. Our results demonstrated that Sirt3, but not the other mitochondrial sirtuins tested, binds to SOD2. In addition, SOD2, but not the other SOD family members tested, binds to Sirt3. Furthermore, we showed that the protein levels of Sirt3 negatively corresponded with the acetylation level of SOD2; as the Sirt3 level decreased, the SOD2 acetylation increased. As previously discussed, the activity of SOD2 is inversely proportional to the acetylation level, thus indicating that Sirt3 regulates the activity of SOD2. However, the down-regulation of SOD2 at both the mRNA and protein levels could contribute to SOD2 activity. On the basis of the results in our study, at 10 months of age, the SOD2 activity of the D-Gal group was significantly decreased compared with the age-matched NS group (P<0.05), while the SOD2 mRNA and protein levels showed no significant difference (P>0.05). Thus, it is reasonable that the SOD2 activity may be regulated by a secondary mechanism that is independent of the SOD2 mRNA and protein levels. At 18 months of age, compared with the age-matched NS group, the SOD2 activity was reduced by 4.50-fold, the SOD2 mRNA expression was reduced by 2.04-fold, the SOD2 protein level was reduced by 2.27-fold, the SOD2 acetylation level was increased by 3.31-fold, and the Sirt3 protein level was reduced by 3.47-fold. Taken together, these results suggested that both the decrease in Sirt3 and SOD2 contributed to SOD2 activity. Consistent with the previous studies [40]-[42], our findings showed that Sirt3 may play an important role in ROS homeostasis by deacetylating SOD2 and regulating the antioxidant activity of SOD2.

In conclusion, this study indicates that Sirt3 is present in the rat central auditory cortex and is decreased in natural aging and D-Gal-induced mimetic aging rats. Thus, studies using the D-Gal-induced aging model may provide new mechanistic insight into central auditory dysfunction in AHL. We propose that decreased Sirt3 may have less ability to deacetylate SOD2, resulting in a reduction in SOD2 activity and an elevation in ROS generation. As a result, oxidative stress accumulated with aging, mitochondria dysfunction, abnormal ultrastructural changes and auditory cortex cell apoptosis occur, which ultimately results in AHL. Because the present approaches focused on the compensation of peripheral dysfunction are costly and have limited efficacy, treatment options aimed at increasing Sirt3 expression will open doors to the management of central presbycusis. Thus, this study may shed light on the pathogenesis of central presbycusis and provide a new target for age-related degenerative diseases in the central nervous system.
