# Correction: Age-Related Decrease in the Mitochondrial Sirtuin Deacetylase Sirt3 Expression Associated with ROS Accumulation in the Auditory Cortex of the Mimetic Aging Rat Model

**DOI:** 10.1371/journal.pone.0098726

**Published:** 2014-05-23

**Authors:** The PLOS ONE Staff

There is an error in Figures 6, 7, and 8, which were swapped during production. Please view the correct figures and the corresponding figure legends here:

**Figure 1:**
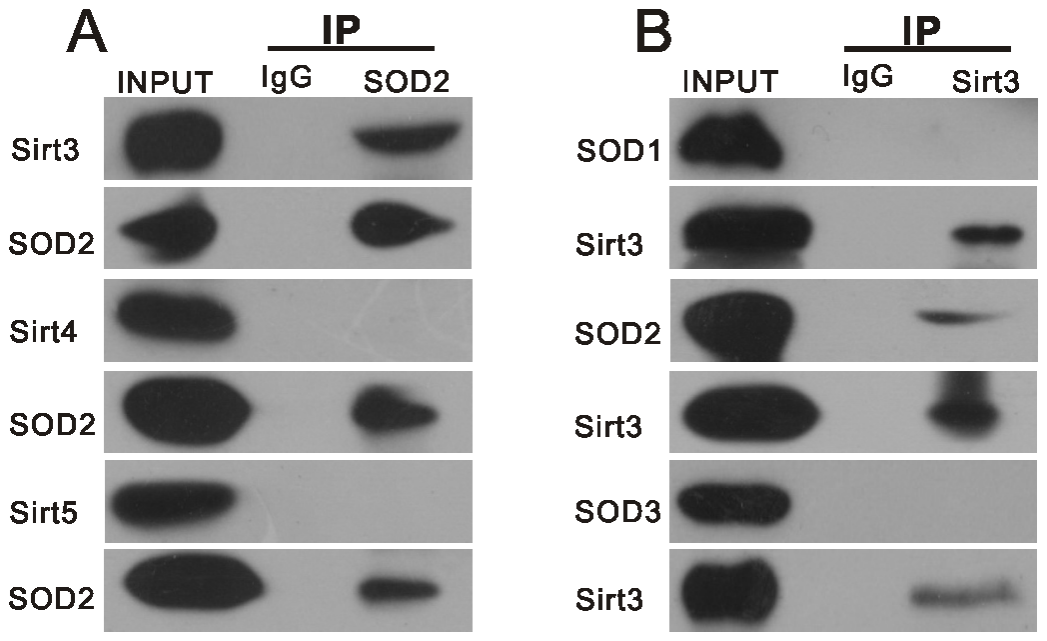
Physical interaction between SOD2 and Sirt3 in the auditory cortex. A. Endogenous SOD2 was immunopurified from the auditory cortex with anti-SOD2 antibody, followed by western blotting with anti-SIRT3, anti-Sirt4 and anti-Sirt5 antibodies. B. Endogenous Sirt3 was immunopurified from the auditory cortex with anti-Sirt3 antibody, followed by western blotting with anti-SOD2, anti-SOD1 and anti-SOD3 antibodies.

**Figure 2:**
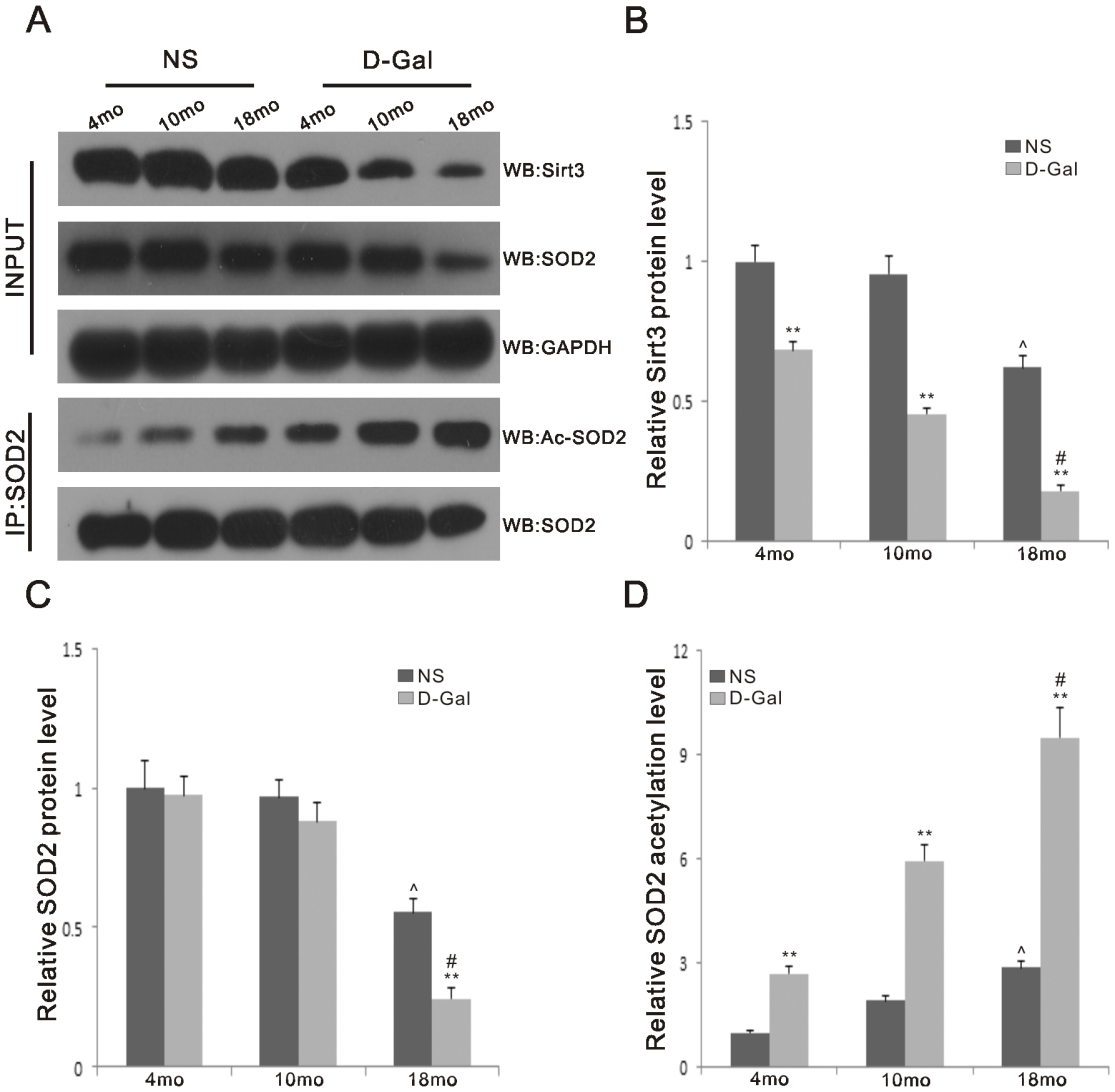
Protein levels of Sirt3 and SOD2 and acetylation levels of SOD2 in the auditory cortex. A. Top panels: Western blotting analysis of Sirt3 and SOD2 in the auditory cortex from the 4-, 10- and 18-month-old rats in the NS and D-Gal groups. GAPDH was used as a reference. Lower panels: Endogenous acetylated SOD2 was isolated by immunoprecipitation with anti-SOD2 antibody followed by western blotting with anti-acetyl-lysine antibody. SOD2 was used as a reference. (n  =  6 per subgroup) B. Quantification of the amounts of total Sirt3 protein (Fig. 7B) from (Fig. 7A).The levels of Sirt3 protein were significantly decreased in the D-Gal groups compared to the NS groups, as well as in the 18-month-old groups compared to the 4-month-old groups. C. Quantification of the amounts of total SOD2 protein (Fig. 7C) from (Fig. 7A). The levels of SOD2 protein was significantly decreased between the 18-month-old D-Gal and NS groups. Significant differences were also found between the 4- and 18-month-old groups. D. Quantification of the amounts of SOD2 acetylation (Fig. 7D) from (Fig. 7A). The levels of SOD2 acetylation were significantly increased in the D-Gal groups compared to the NS groups, as well as in the 18-month-old groups compared to the 4-month-old groups. **Significantly different from the NS groups (P<0.01). ∧Significantly different from the 4-month-old NS group (P<0.01). #Significantly different from the 4-month-old D-Gal group (P<0.01).

**Figure 3:**
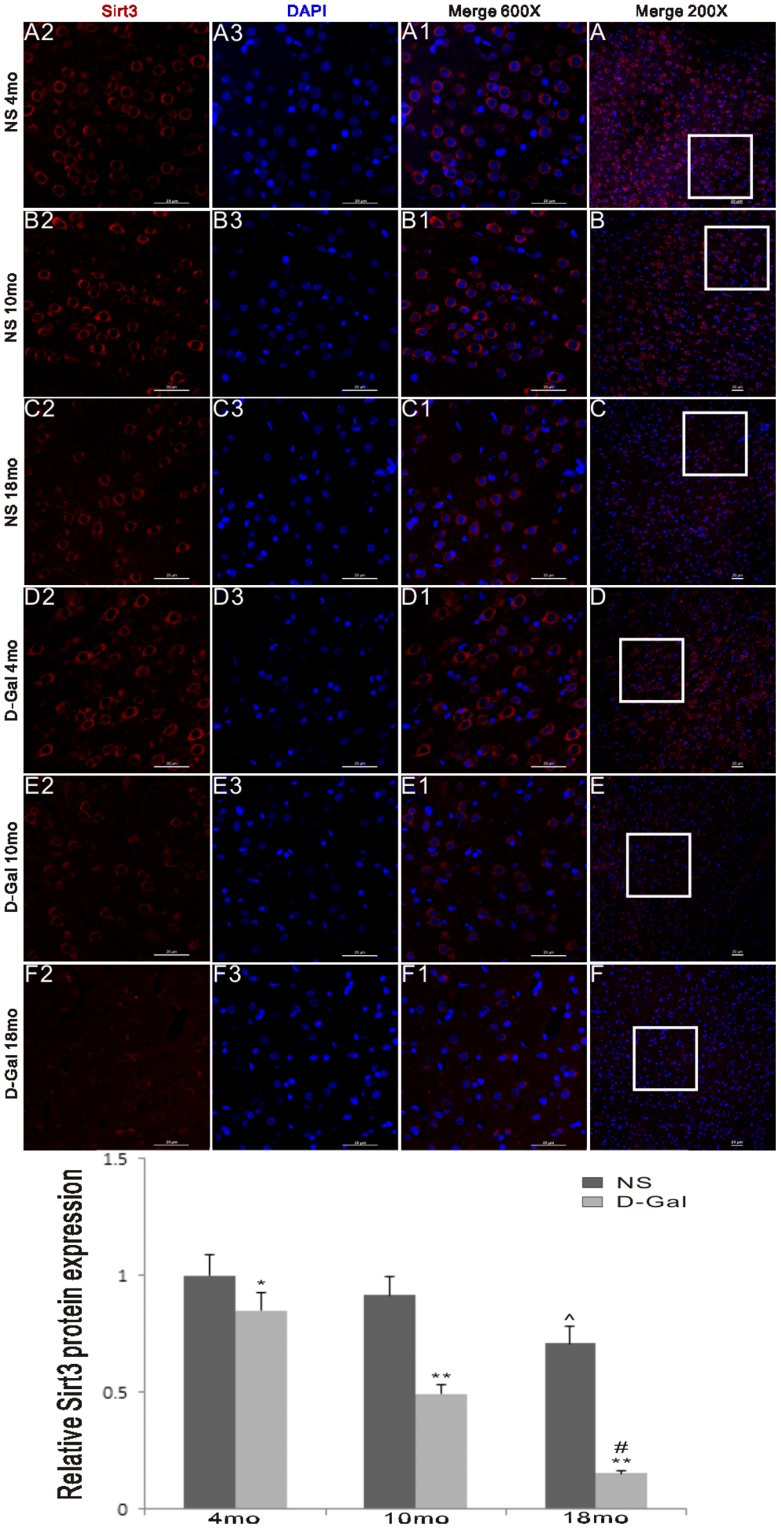
Sirt3 protein expression in the auditory cortex. An immunofluorescence assay was used to measure the effects of age and D-Gal on Sirt3 protein expression in the auditory cortex. The levels of Sirt3 protein expression in the D-Gal groups were significantly lower compared to the NS groups. The levels were also decreased in the 18-month-old groups compared to the 4-month-old groups.
